# Does Diversion in Poorly Functioning Obstructed Kidneys in Adults Favors Reconstructive Surgeries Over Ablative Procedures? A Prospective Study

**DOI:** 10.7759/cureus.10124

**Published:** 2020-08-29

**Authors:** Sidhartha Kalra, Ketan Mehra, Kaliyaperumal Muruganandham, Lalgudi N Dorairajan, Ramanitharan Manikandan, Halanaik Dhanapathi, Sreerag Sreenivasan Kodakkattil

**Affiliations:** 1 Urology and Renal Transplantation, Jawaharlal Institute of Post Graduate Medical Education and Research, Pondicherry, IND; 2 Nuclear Medicine, Jawaharlal Institute of Post Graduate Medical Education and Research, Pondicherry, IND

**Keywords:** poorly functioning kidneys, nephrectomy, percutaneous nephrostomy, pelviureteral junction obstruction, pyeloplasty

## Abstract

Objective

In obstructed poorly functioning kidneys, management depends on the recovery potential of the kidney. Some kidneys have good recovery capability and diversion may unfold the real condition of the kidney. This study evaluated whether pre-operative drainage for six weeks results in improvement of renal function in unilateral obstructed poorly functioning kidney with split renal function (SRF) less than 20%.

Methods

This was a prospective interventional study conducted between March 2013 and December 2015. All patients between 15 and 65 years, with unilaterally obstructed kidney with SRF ≤20% underwent percutaneous nephrostomy (PCN) drainage for six weeks. Patients having post-drainage SRF of ≥15% and per day urine output from PCN > 400 ml were considered for the reconstructive procedure. Nephrectomy was performed in cases with SRF <15% after considering patient preferences.

Results

Twelve of 17 patients had improvement in SRF; four had no change while one had a decrease in SRF after drainage. The mean improvement in glomerular filtration rate (GFR) and SRF was 1.4 ml/min and 3%, respectively (P = 0.08). Three out of seven patients with SRF of ≥15% showed an improvement of 5% or more while none of the patients with SRF <15% had such an improvement. Eight patients had final SRF <15% and underwent nephrectomy. Factors such as pre-existing SRF, duration of symptoms, kidney size, transverse pelvic diameter, 24-hour urinary output, and etiology for obstruction were not significant in predicting functional improvement.

Conclusion

Diversion and decompression of poorly functioning kidneys do not result in a significant functional improvement in obstructed kidneys with SRF <15%.

## Introduction

Poorly functioning obstructed kidney in adults is a common problem encountered by the urologist. Surgical management of these kidneys is usually based on the differential renal function calculated on the diuretic renogram [1]. The decision to remove or preserve a poorly functioning obstructed kidney depends on its function. It is recommended to reassess the function of these kidneys at four to six weeks after the relief of obstruction. However, this recommendation is based on an animal study conducted by Kerr in 1954 [2]. There have been conflicting views addressing this issue in humans. Few reports consider that there is a functional recovery after drainage while others are of the view that there is no chance of improvement of function in poorly functioning obstructed kidneys.

There is still a considerable debate as at what level of split renal function (SRF) should a kidney preserved. Few authors consider SRF of less than 10% as the threshold for reconstruction [3]. In contrast, few authors opine that drainage in patients with SRF <30% may not have significant improvement [4]. At our institution, we routinely consider a cutoff of SRF <15% for the ablative procedure while the decision for SRF between 15% and 20% is left to individual surgeon’s discretion and patient preferences. Limited literature is available on pre-operative factors that could predict functional improvement in patients with the affected renal unit after drainage [5]. Additionally, there has been inconsistency in the accurate measurement of SRF in poorly functioning hydronephrotic kidneys and extrapolating these results to a functional outcome [6].

The present study was designed to evaluate whether drainage for six weeks results in improvement of renal function in unilateral obstructed poorly functioning kidney with SRF <20%. Factors associated with functional recovery and correlation of glomerular function (measured by diethylene triamine penta-acetic acid [DTPA] scan [Gamma camera method]) and creatinine clearance (CRCL) were also assessed.

## Materials and methods

This was a prospective interventional study conducted at the Department of Urology, Jawaharlal Institute of Postgraduate Medical Education and Research (JIPMER), Puducherry between March 2013 and December 2015. All patients presenting with unilateral obstructed poorly functioning kidney, SRF ≤20% measured by technetium-99m DTPA (99mTc DTPA) and age ranged from 15 to 65 years were included in this study. Patients with bilateral obstruction, obstruction in a solitary kidney, malignant obstruction, pyonephrotic kidneys, and deranged renal functions were excluded from the study.

Baseline demographic characteristics including age, sex, etiological factor, and duration of symptoms were recorded. Eligible patients underwent a renal function test and abdominal ultrasonography to check for kidney size and dilatation of the pelvicalyceal system at presentation. The renal function tests were performed by a 99mTc DTPA scan using the gamma camera method.

Ultrasound-guided percutaneous nephrostomy (PCN) was performed under local anesthesia on obstructed moiety as a daycare procedure. Any associated complications such as infection, bleeding requiring admission, PCN blockade, or slipping were recorded and treated. Patients were on regular follow-up and at six weeks, a repeat DTPA scan was performed to note any change in renal function after drainage, and subsequently, patients were admitted for definitive management. CRCL of the drained moiety was estimated using the formula CrCl = (UCr x V)/SCr (serum creatinine-adjusted for body surface area), where UCr represents 24-hour urinary creatinine of the drained moiety and V represents 24-hour urine volume from PCN.

Patients with post-drainage SRF ≥15% and per day urine output post-PCN of more than 400 ml were considered for the reconstructive procedure. Nephrectomy was performed if SRF was less than 15%. Various factors predicting functional recoveries such as age, pre-existing SRF, duration of symptoms, renal morphometric parameters, daily PCN urinary output, and etiology were recorded and analyzed. The correlation between glomerular filtration rate (GFR) measured by DTPA scan and urinary CRCL was also assessed.

The sample size of 22 was considered sufficient to detect a difference in SRF of 10% and an SD of 12.6 from baseline. Calculations were based on α of 0.01 and power of 80%. The statistical analysis was performed using professional statistics package EPI Info 7.0 version (CDC, Atlanta, USA) for windows. Descriptive data were represented as mean (SD) for numeric variables, percentages, and proportions for categorical variables. Wilcoxon’s rank test was used to compare SRF/GFR of the pre- and post-drainage poorly functioning kidney. Spearman’s correlation coefficient was used to assess the association between improvement in SRF with age, daily PCN urinary output, kidney size and transverse parenchymal diameter, and also the correlation between GFR calculated on DTPA and by urinary CRCL method. Simple linear regression was applied when the correlation was found to be statistically significant. Independent T-test was used to assess the significance of various factors in predicting functional recovery. Values of p < 0.05 was considered statistically significant.

## Results

A total of 21 patients who fulfilled the inclusion and exclusion criteria were enrolled in the study. Two patients withdrew their consent and two patients got operated at a different institution during the follow-up period of six weeks for personal reasons. Hence, 17 patients (80%) who completed the intervention were analyzed.

Of the total 17 patients, 10 (58.52%) were males and seven (41.18%) were females (Table 1). The overall mean (SD) age was 36.12 (12.16) years. The mean duration symptoms were 9.81 months, the kidney size was 11.97 cm, and the mean transverse pelvic diameter was 5.14 cm. The mean serum creatinine was 1.08 mg/dL. A total of 10 (58.82%) patients had primary pelviureteric junction obstruction (PUJO), four had calculus disease, and three had upper ureteric stricture as the cause of obstruction (Table 2).

**Table 1:** Demographics and clinical characteristics at admission SD: standard deviation.

Characteristics	N = 17
Age in years (SD)	36.12 (12.16)
Male (%)	10 (58.82)
Female (%)	7 (41.18)
Duration of symptoms in months (SD)	9.81 (5.54)
Kidney size in cm (SD)	11.97 (2.4)
Transverse Pelvic diameter in cm (SD)	5.14 (3.521)
Mean serum creatinine in mg/dL (SD)	1.08 (0.17)

**Table 2:** Etiology of obstruction PUJO: pelviureteric junction obstruction.

Diagnosis	Number (%)
PUJO (%)	10 (58.82)
Renal and upper ureteric calculi (%)	4 (23.53)
Upper ureteric stricture (%)	3 (17.65)

The PCN was successfully performed in all 17 patients and none of the patients had urinary tract infection or bleeding after PCN. The PCN drainage was kept for six weeks and then definitive surgery was performed.

The mean (SD) GFR pre-drainage calculated by DTPA scan was 8.21 (4.5) ml/min and the SRF was 13.0% (4.35). The mean (SD) GFR post-drainage was 9.60 (5.97) ml/min and the SRF was 16.12% (7.35). Twelve (70.59%) of 17 patients had improvement in SRF; four (23.53%) had no change while one (5.88%) had a decrease in SRF after drainage. The mean improvement in GFR and SRF was 1.4 ml/min and 3%, respectively. This improvement in GFR and SRF was statistically significant (p = 0.008).

**Figure 1:**
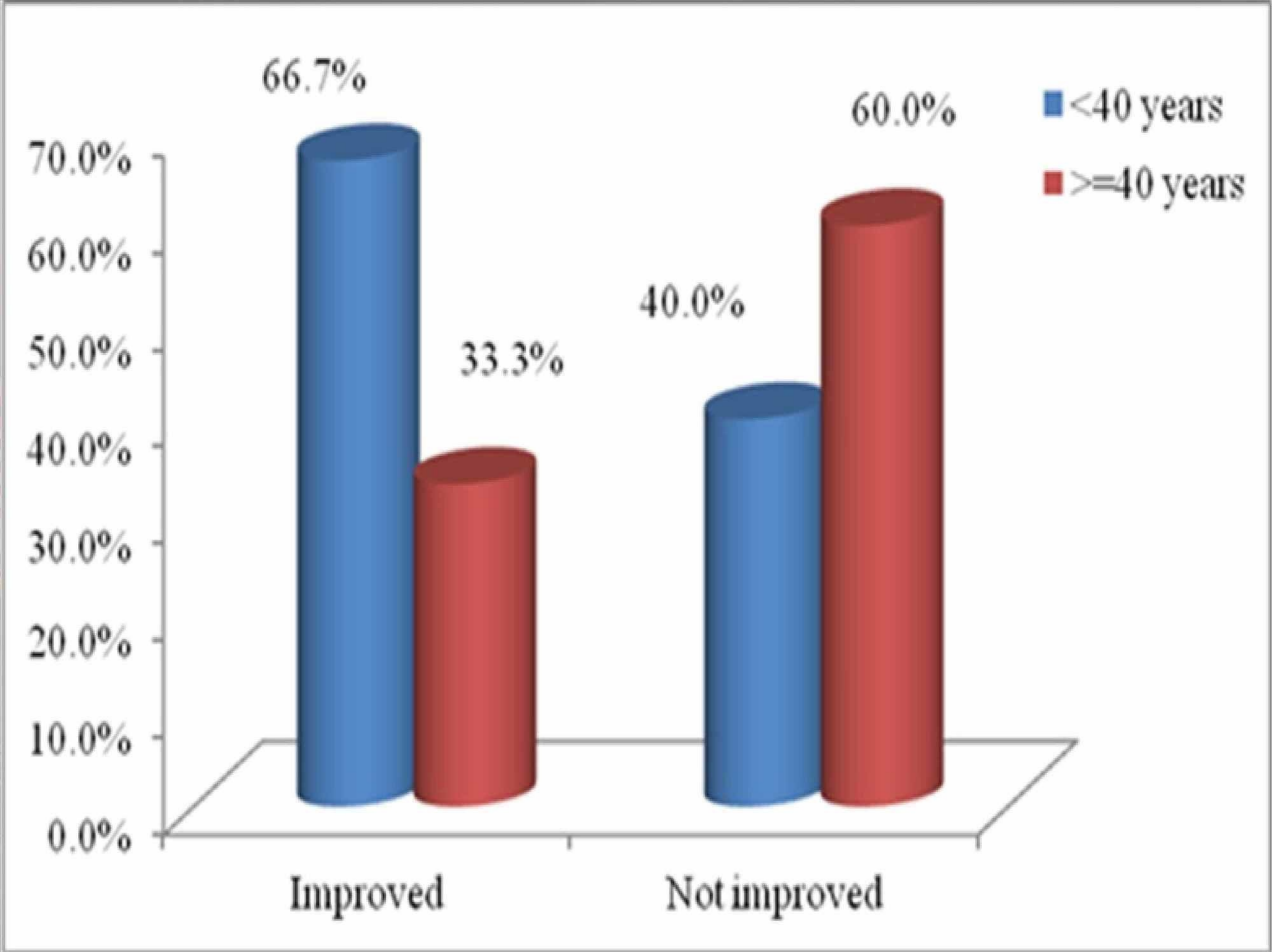
Graph showing age and improvement in SRF SRF: split renal function.

The mean (SD) age in patients showing improvement in SRF was 34.58 (7.17) compared to 39.80 (20.63) years in patients showing no improvement. There was a negative correlation between the two, however, statistically insignificant (p = 0.438; Figure 1). The mean (SD) urinary drainage from PCN was 773.53 (537.10) ml. Twelve patients with improvement in SRF had the mean output of 883.33 ml, while five patients with no improvement in SRF had the mean drainage of 510. However, this difference in urine output production in patients showing improvement versus no improvement was not statistically significant (p = 0.20; Table 3).

**Table 3:** Comparison of patients with improvement versus no improvement after PCN PCN: percutaneous nephrostomy.

Parameters	Improvement	No improvement	P-value
Patients, number	12	5	0.008
Age (years)	34.58 (7.17)	39.80 (20.63)	0.438
Duration of symptoms (months)	9.92 (5.91)	9.5 (5.00)	0.902
Kidney size (cm)	12.23 (2.42)	11.34 (2.70)	0.512
PCN urinary output (mL)	883.33 (562.60)	510 (400.63)	0.201

Ten patients with initial SRF of less than 15% showed a mean (SD) percent improvement in SRF of 14.1% (24.33) contrary to 36.6% (33.31) mean percent change in SRF seen in seven patients with initial SRF more than 15%. This difference was not statistically significant (p = 0.127)

**Figure 2:**
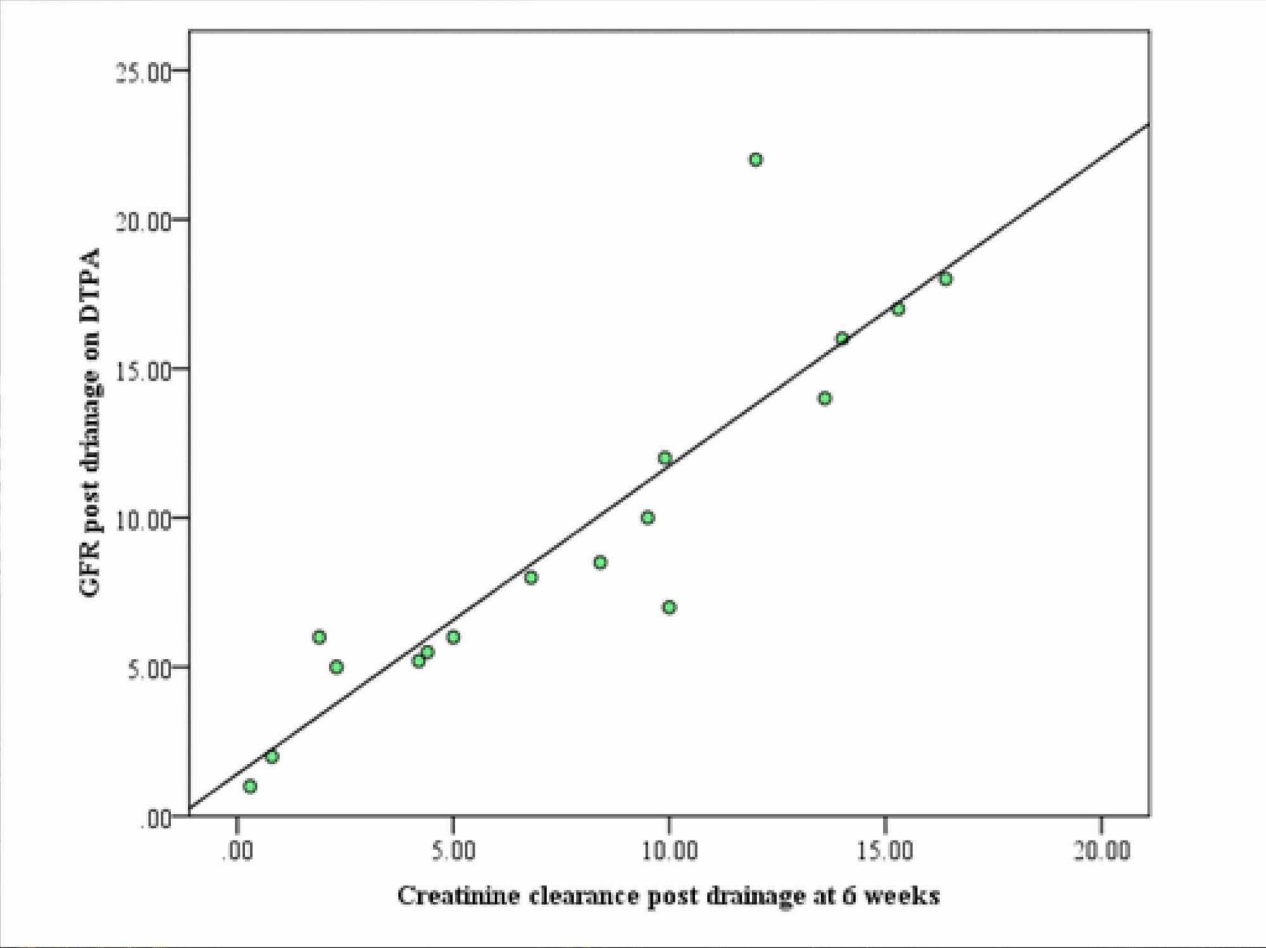
Graph showing correlation between GFR by CRCL and DTPA scan GFR: glomerular filtration rate, CRCL: creatinine clearance, DTPA: diethylene triamine penta-acetic acid.

The mean (SD) GFR calculated by urinary CRCL method was 7.92 (5.22) compared to 9.60 (5.97) calculated on DTPA. An almost perfect positive correlation was found between GFR calculated by urinary CRCL and DTPA method with a correlation coefficient of 0.93 approaching close to the value of 1 (Figure 2).

No adverse events were recorded in the form of bleeding, infection PCN blockage, and slippage in any participants of the study. At a one-year follow-up interval, none of the patients who underwent the reconstructive procedure had any further deterioration in renal function and required any additional procedure.

## Discussion

This prospective study evaluated whether drainage for six weeks results in improvement of renal function in unilateral obstructed poorly functioning kidney with ≤20%. The results showed that PCN drainage did not result in a significant functional improvement and clinical outcome in obstructed kidneys with SRF ≤15%.

Surgical management of poorly functioning obstructed kidneys in adults is usually based on the differential renal function calculated on the diuretic renogram. Although drainage decreases hydronephrosis and improves the excretory pattern, a significant increase in differential renal function occurs only in some patients. Various factors that could predict the functional recovery of these kidneys after drainage are not conclusively defined in the literature. With the advancement in the field of diagnostic imaging, most of these cases are detected early in life. Thus, most of the studies concerning this issue are focused on pediatric patients rather than adults.

Various studies have shown different independent predictors for the improvement and resolution of renal function. In a previous study, Gupta et al. analyzed the usefulness of PCN drainage in patients with ureteropelvic junction obstruction having SRF <10% (n = 20) [7]. Twelve of 17 kidneys showed improvement in function after drainage while the rest of the five units did not show any functional recovery. Gupta et al. concluded that drainage in such kidneys may help in differentiating kidney which is likely to improve and not all such kidneys should be removed [7]. In another study, Ransley et al. included 112 patients (142 kidneys) with prenatally diagnosed hydronephrosis [8]. Out of these nine kidneys had a poor function on renogram at three months and underwent pigtail drainage. Three of these nine kidneys recovered with sufficient function and subsequently underwent pyeloplasty. Ransley et al. recommended attempting drainage in cases where SRF is <20% but >10% [8]. However, kidneys having SRF <10% should be directly considered for an ablative procedure without trying drainage.

Overall, most of these studies were conducted in the pediatric age group where the kidneys have the potential for development. Mayor et al. in their study found an association of early drainage and improvement in function with age [9]. In another study Chandrasekharam et al. also concluded that functional recovery was significantly higher in patients less than one year of age undergoing pyeloplasty [10]. In contrast, MacNeily et al. did not find the patient’s age as a predicting factor in functional recovery [11].

Ortapamuk et al. studied the improvement in differential function in adult obstructed kidneys with PUJO [4]. Patients were distributed into two groups according to baseline differential function. A total of 22 patients had an initial SRF of >30% (group 1) while 10 patients had SRF <30% (group 2). Improvement in SRF was found to be significant when there was a 5% increment in differential function over the baseline. They found a statistically significant improvement in differential function in group 1 with differential function more than 30 compared to the other group. However, no significant correlation between functional recovery with ultrasonography findings, clinical presentation, and age among the two groups was found. They concluded that improvement in SRF in adult obstructed kidney depends on the initial level of SRF and patients with differential function <30% may not benefit much from drainage procedure.

Recently, Zhang et al. retrospectively analyzed 53 patients with unilateral PUJO with SRF <10% who underwent PCN as the drainage procedure and patients were considered as improved if there was a functional recovery of >10% and daily PCN drainage was >400 ml [3]. At six weeks of drainage, 30 of 53 (56.6%) patients showed an improvement in the mean GFR and SRF. The before and after PCN, the mean (SD) GFR was 3.57 (2.79) and 14.05 (5.42) ml/min/1.73 m^2^, and the mean (SD) SRF was 4.53% (3.21) and 16.07% (5.49). Authors concluded that such poorly functioning obstructed kidneys should be considered for drainage especially in young adults (age <35 years). Moreover, diuretic renography with 99mTc-DTPA was not a reliable assessment of renal function in these obstructed kidneys.

In the present study, we evaluate the functional recovery after drainage in the unilaterally obstructed kidney with SRF ≤20%. In this study, 12 of 17 patients (70%) showed a statistically significant improvement in SRF after drainage. The mean (SD) pre- and post-drainage SRF was 13.0% (4.35) and 16.12% (7.35), respectively. However, only nine of these 12 patients had the final SRF ≥15% and underwent the reconstructive procedure. The rest of the three patients despite showing percent change in SRF (post-pre-drainage/pre-drainage SRF) had final SRF of <15% and who had nephrectomy. In total, 10 out of 17 patients (53%) who had an initial SRF <15% showed a mean (SD) percent improvement in SRF of 14.1% (24.32) while seven patients with initial SRF >15% had 36.6% (33.3) improvement in SRF. This difference was statistically insignificant (p=0.13).

However, taking into account the criteria of 5% improvement in SRF as significant, three out of seven patients (42%) with initial SRF of >15% showed such improvement while none of the patients with SRF <15% showed improvement of 5% or more. A negative correlation was observed between age and functional recovery although statistically insignificant. The mean (SD) age in patients with improvement in SRF was 34.58 (7.17) years in comparison to 39.80 (20.63) years in patients with no functional recovery. Zhang et al. in their study found a statistically significant association of improvement in SRF with age [3]. In this particular study, 24 out of 29 adults with a mean (SD) age of 28.28 (4.88) years (82.8%) showed improvement in SRF; however, only six of 24 (25%) patients with a mean (SD) age 51.21 (10.69) years showed improvement.

A positive correlation was observed between the percent change in SRF and 24-hour urinary output from PCN. However, the difference in urinary output from PCN among patients having functional improvement versus those with no change in function was statistically insignificant. Thus, PCN urinary output as an independent factor may not be adequate for predicting the salvage ability of these kidneys and assessment of a repeat differential function could be necessary before considering definitive management. Other factors such as duration of symptoms, kidney size, transverse pelvic diameter, and etiology were not found to be significant in predicting the functional improvement after drainage in the present study. This observation was similar to a previous report by Ortapamuk et al. [4].

The mean (SD) GFR estimation by CRCL at six weeks post-drainage was 7.92 (5.22) ml/min which was slightly less compared to 9.60 (5.97) ml/min calculated on DTPA scan. However, an almost perfect positive correlation was seen between the two on statistical analysis with a correlation coefficient of 0.93 reaching the value of 1. A similar association was reported by Ulibarri et al. who calculated GFR in post-renal transplant patients by DTPA, CRCL method, and GFR-estimating equations [12]. They observed a significant correlation between GFR estimated by the CRCL method and DTPA scan. DTPA scan although not a definitive assessment of SRF and GFR in such poorly functioning kidney can still be a fair guide of renal function.

Pre-existing SRF was found to be statistically insignificant in predicting the functional recovery in our study. However, it was seen that most of the patients with initial SRF <10% did not show a significant improvement after drainage with only two out of 10 patients (20%) reaching the salvageable limit of 15%. Both of them had an initial SRF of 14%. Although a statistically significant mean improvement of 3% in SRF and 1.4 ml/min in GFR was seen in this study, it was not sufficient to extrapolate to a clinical improvement.

Nayyar et al. in 2016 studied 32 patients with SRF <20% who underwent pyeloplasty for pelviureteral junction obstruction [13]. In their study, the mean follow-up was 26.8 months and none of the patients required any re-intervention for obstructive drainage, intractable pain, or deteriorating function. There was a success rate of 93.7% and 40.6% showed significant improvement in SRF which was >5% over perioperative period. In their study, only one patient (3.1%) had the deterioration of function. They concluded that pyeloplasty provided a high rate of success both functional and morphologically in poorly functioning kidney with pelviureteral junction as the etiology. This study was different from our study as it considered only pelviureteral junction obstruction as the etiology and also they proceeded with the definitive surgery instead of pre-operative drainage.

Thus, the crux of the problem not only lies in accurately measuring the differential function of these poorly functioning obstructed kidney but also in deciding the definitive treatment. Since, subjecting these kidneys to a reconstructive procedure has the future risk of infection, the persistence of symptoms and a further decrease in function with time.

## Conclusions

PCN drainage did not result in a significant functional improvement and clinical outcome in obstructed kidneys with SRF <15%. Diuretic renography with a DTPA scan provided a fair estimate of function in these obstructed units. An almost perfect positive correlation of GFR estimation by DTPA scan and CRCL method was observed. A negative correlation was seen between age and functional recovery, however, statistically insignificant. Factors such as duration of symptoms, kidney size, transverse pelvic diameter, and etiology are not predictive of functional recovery after drainage. Further prospective studies with a larger sample size are warranted to confirm these results.
